# A Metagenomic Comparison of the Colostrum Microbiome in Bulgarian Mothers by Delivery Mode: A Pilot Study

**DOI:** 10.3390/microorganisms14010184

**Published:** 2026-01-14

**Authors:** Daniela Mollova, Vesselin Baev, Tsvetomira Borisova, Mariya Rusinova, Ilia Iliev

**Affiliations:** 1 Department of Biochemistry and Microbiology, Faculty of Biology, University of Plovdiv, Tzar Assen 24, 4000 Plovdiv, Bulgaria; iliailiev@uni-plovdiv.bg; 2 Department of Molecular Biology, Faculty of Biology, University of Plovdiv, Tzar Assen 24, 4000 Plovdiv, Bulgaria; baev@uni-plovdiv.bg; 3 Children’s Nutrition Complex with Human Milk Bank, Sava Mihaiylov St. 57, 1309 Sofia, Bulgaria; cv_borisova@abv.bg (T.B.); dr_maria_rusinova@abv.bg (M.R.)

**Keywords:** colostrum, delivery mode, microbiome, infant

## Abstract

Colostrum harbors a highly diverse microbial community, predominantly composed of genera such as *Staphylococcus*, *Streptococcus*, *Lactobacillus*, *Bifidobacterium*, and *Enterococcus*. The composition and diversity of this microbiota are influenced by maternal factors—including age, body mass index, lactation activity, stress levels, and gestational diabetes—as well as external factors such as mode of delivery, antibiotic exposure, diet, and geographic location. This microbial community plays a critical role in maternal and neonatal health by contributing to early gut colonization, supporting digestion, promoting immune system development, and protecting against pathogenic microorganisms through mechanisms such as antimicrobial peptide production by lactic acid bacteria. The primary aim of this study was to evaluate the impact of mode of delivery on colostrum microbiota by comparing mothers who delivered vaginally with those who underwent cesarean section. Colostrum samples from 15 mothers were subjected to DNA extraction, high-throughput sequencing, and bioinformatic analyses to characterize microbial composition and predicted functional profiles. Although substantial inter-individual variability was observed, no statistically significant differences were detected in overall microbial diversity or community structure between the two delivery groups. However, distinct bacterial taxa and functional characteristics were identified that were specific to each mode of delivery, suggesting subtle delivery-related influences on colostrum microbiota composition.

## 1. Introduction

Human breast milk is considered the optimal feeding method for newborns and has been widely acknowledged for its advantages in promoting infant health, growth, and development. The World Health Organization (WHO) and UNICEF advise that breastfeeding should begin within the first hour after birth and that infants should be exclusively breastfed for the initial six months [1]. Human milk is a complex biological substance and serves as the most essential nutritional source during early infant growth and development [2]. The bioactive components of human milk adapt in response to the nutritional and immune needs of the breastfeeding infant. Understanding the components of breast milk and their roles can enhance clinical practice, optimize infant feeding, and deepen our understanding of infants’ immune responses to infections and vaccinations [3]. Human milk is produced and secreted by the mammary glands and delivered to the nursing infant to provide essential nutrition, immune protection, and developmental support [4]. Beyond its role as an optimal source of nutrients, human milk also contains a variety of bioactive components, including immune and stem cells, interleukins, antibodies (IgA and IgG), hormones, oligosaccharides, peptides, and free amino acids (FAAs), among others [5]. Research has further demonstrated that human milk harbors its own microbiota, including diverse bacterial species, in lactating mothers. It has been reported that human milk is a major source of bacteria for the gut of breastfed infants, with a baby consuming around 800 mL of milk per day, potentially ingesting between 1 × 10^5^ and 1 × 10^7^ bacteria daily [6]. The source of bacteria in breast milk remains unclear, but their presence is associated with the perinatal period, beginning in the third trimester of pregnancy and continuing throughout lactation [7]. Some studies suggest that the human milk microbiota may originate from the mother’s skin, the infant’s oral cavity during breastfeeding, or the mother’s gut through the entero-mammary pathway [8]. Additionally, research has identified a commensal microbiota within human breast tissue [9], indicating that certain microbes may reside in the breast tissue and potentially colonize the milk ducts. However, the specific impact of these microbes on both short- and long-term neonatal health remains unclear. Colostrum samples have been found to exhibit greater microbial diversity compared to mature milk. The stage of lactation has been identified as a factor that affects the composition of milk microbiota. In the early stages, the microbial community primarily comprises *Weissella*, *Leuconostoc*, *Staphylococcus*, *Streptococcus*, and *Lactococcus* species. As lactation progresses, microbiota shifts to include higher abundances of *Veillonella*, *Prevotella*, *Leptotrichia*, *Lactobacillus*, and *Streptococcus* species, along with increasing levels of *Bifidobacterium* and *Enterococcus* species [10]. The way a baby is delivered influences the composition of microbiota in human milk. Colostrum and milk from vaginal deliveries typically exhibit greater microbial diversity and higher levels of *Bifidobacterium* and *Lactobacillus* species, whereas milk from cesarean deliveries often shows the opposite pattern [11]. However, some studies have found no differences in milk microbial profiles associated with gestational age, delivery method, or infant sex [12]. Gestational age also affects breast-milk microbiota, with notable differences between term and pre-term deliveries. For instance, colostrum from term deliveries tends to have lower Enterococcus counts, and milk shows higher *Bifidobacterium* counts. Additionally, maternal health conditions such as obesity, celiac disease, or HIV infection can alter the composition of milk microbiota [11]. While the composition of the human milk microbiome differs across populations, several dominant genera have been consistently identified in early lactation, including *Staphylococcus*, *Streptococcus*, *Pseudomonas*, *Acinetobacter*, *Bifidobacterium*, *Mesorhizobium*, *Brevundimonas*, *Flavobacterium*, and *Rhodococcus* [13,14]. Growing research highlights the important role of human milk (HM) microbiota in shaping gut colonization and supporting infant development [15,16,17]. Kumar et al. demonstrated that the composition of human milk microbiota in healthy mothers varies notably across countries, including Spain and Finland in Europe, South Africa in Africa, and China in Asia [18]. Across the different countries, 23 phylotypes were commonly present in HM microbiota at the family level. *Lactobacillaceae* were found only in Finnish samples, Bifidobacteriaceae were exclusive to South African samples, and Enterococcaceae were not detected in Chinese HM. Among women who gave birth vaginally, Spanish mothers had the highest levels of *Bacteroidetes*, whereas Chinese mothers showed elevated levels of *Actinobacteria.* Overall, women from Spain and South Africa exhibited significantly greater abundance of bacterial genes involved in carbohydrate, lipid, and amino acid metabolism [18]. The composition of human milk changes not only with the stages of lactation but also in relation to the environment in which the mother and infant live. This highlights the need to study the human milk microbiota and its role across diverse regions with varying ethnic and cultural backgrounds worldwide [19]. Several short-term studies have examined the human milk microbiome during early lactation, identifying hundreds of bacterial species [20,21,22]. These studies, which focused on small groups of healthy women within the first month after giving birth, revealed noticeable differences in the core bacterial genera reported. It is also well established that breast milk composition is highly individual and influenced by multiple factors, including maternal diet, genetics, health status, antibiotic use, mode of delivery, as well as demographic and environmental conditions. The primary aim of this study was to comprehensively evaluate the impact of mode of delivery on the colostrum microbiota by comparing microbial composition, diversity, and predicted functional profiles between mothers who delivered vaginally and those who underwent cesarean section.

## 2. Materials and Methods

### 2.1. Subjects and Sample Collection

The Clinical Research Ethics Committee of the University of Plovdiv “Paisii Hilendarski” approved this study (Approval No. 8/2 April 2025). Written informed consent was obtained from all participants, and the study was conducted in accordance with applicable guidelines and regulations. Of the 20 full-term breastfeeding women who enrolled in the study, 15 provided a milk sample at one point. Participants were healthy breastfeeding women who had delivered at full term, either vaginally or by Cesarean section. Eligibility criteria included a gestational age of at least 37 weeks, a birth weight greater than 1500 g, and no maternal antibiotic treatment course during pregnancy or in the postpartum period (single prophylactic peri-operative doses after cesarean delivery were not considered exclusionary). The clinical characteristics of the participants are shown in Supplementary Information Table S1. Mothers were instructed to rinse the breast with water, express 20–25 mL of milk into a provided sterile container, and refrigerate it at 4 °C until collection and transport to the laboratory. All samples were collected within the first three days after birth, and the collection was carried out in one go, not in separate portions. It should be noted that the onset of colostrum production is strictly individual for each woman. The milk samples were processed within 24 h of donation.

### 2.2. DNA Extractions and Sequencing

Microbial DNA was extracted from 15 breast milk samples using a modified protocol from the QIAamp DNA Microbiome Kit (Qiagen, Manchester, UK). Briefly, milk samples were subjected to initial centrifugation at 4000× *g*   30 min at 4 °C, the fat layer was removed with a sterile cotton swab (Thermo Fisher Scientific, Inc., Waltham, MA, USA), and the supernatant was discarded. Cell pellets were washed twice with phosphate-buffered saline (Sigma Aldrich, Saint Louis, MO, USA) and treated with 90 µL of 50 mg/mL lysozyme (Sigma Aldrich), followed by incubation at 55 °C  ×  15 min. Samples were subsequently treated with 28 µL of 20 mg/mL proteinase K (Qiagen, UK) and incubated further at 55 °C for 15 min, followed by using the QIAamp DNA Microbiome Kit protocol. The V3–V4 hypervariable region of the 16S rRNA gene was amplified and sequenced using the NovaSeq Illumina platform with a 2 × 250 bp paired-end (PE) read at Novogene (Novogene Europe, Cambridge, UK). The amplicon size distribution was qualitatively checked with a 2100 Bioanalyzer (Agilent Technologies, Santa Clara, CA, USA). Operational taxonomic units (OTUs) were picked and clustered using the QIIME (v1.9.1) pipeline, and taxonomies were assigned based on the SILVA (v132) database at a 97% identity cutoff value [23]. OTU abundance information was normalized using a standard of sequence number corresponding to the sample with the fewest sequences. Downstream alpha (α) and beta (β) diversity analyses were performed based on this output-normalized data. Community alpha diversity indices were calculated using QIIME (v1.9.1). Unweighted pair group method with arithmetic mean (UPGMA) clustering was performed as a type of hierarchical clustering method to interpret the distance matrix using average linkage and was conducted using QIIME (v1.9.1). We used the Phylogenetic Investigation of Communities by Reconstruction of Unobserved States (PICRUSt) bioinformatics software package https://huttenhower.sph.harvard.edu/picrust/ (accessed on 7 January 2026) for predicting metagenomic functions based on Marker genes (such as 16S rRNA). The function prediction based on the KEGG database can be conducted according to 16S sequencing data.

### 2.3. Statistical Analyses

The statistical analysis of results obtained was performed using the SPSS v.26 software (SPSS Inc., Chicago, IL, USA). All experiments were carried out in duplicate. The different comparisons were analyzed by univariate analysis of variance (ANOVA). Statistical significance was accepted as a *p*-value < 0.05. We used R software version 4.4.3 (developed at Bell Laboratories by John Chambers and colleagues) for statistical computing and graphics.

## 3. Results

Our study aimed to characterize the colostrum microbiome of healthy lactating women who had delivered vaginally or by C-section. We collected samples from 15 healthy women in the first few days after delivery. All women signed informed consent and completed a survey with additional questions. The survey results are summarized in Table 1. Mothers’ ages ranged from 21 to 41 years. Almost half of the women we sampled gave birth vaginally, and the other half by C-section. The gestational week of all births is over 37 weeks.

**Figure 1:**
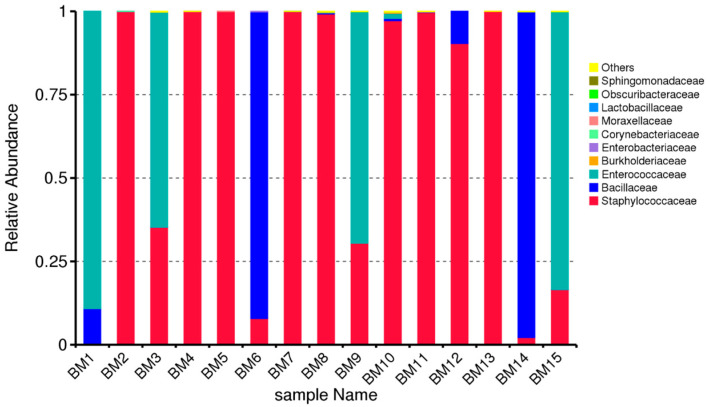
Relative abundance of bacterial families in breast-milk samples (BM1–BM15).

Based on this indicator from the questionnaires, we divided the samples into two main groups: G1 (from mothers who gave birth vaginally) and G2 (from mothers who gave birth by cesarean section). Figure 1 presents the results of the taxonomic distribution of the colostrum samples. A fairly large distribution of the family *Staphylococcaceae* is observed in the individual samples. No presence of this family was reported in sample BM1, and in samples BM6, BM14, and BM15, the presence was significantly low. The figure suggests that: The breast-milk microbiome is generally low-diversity, with a single dominant taxon in most samples, and there is sample-to-sample variation, with a few milk samples showing higher contributions from Gram-negative families.

**Figure 2:**
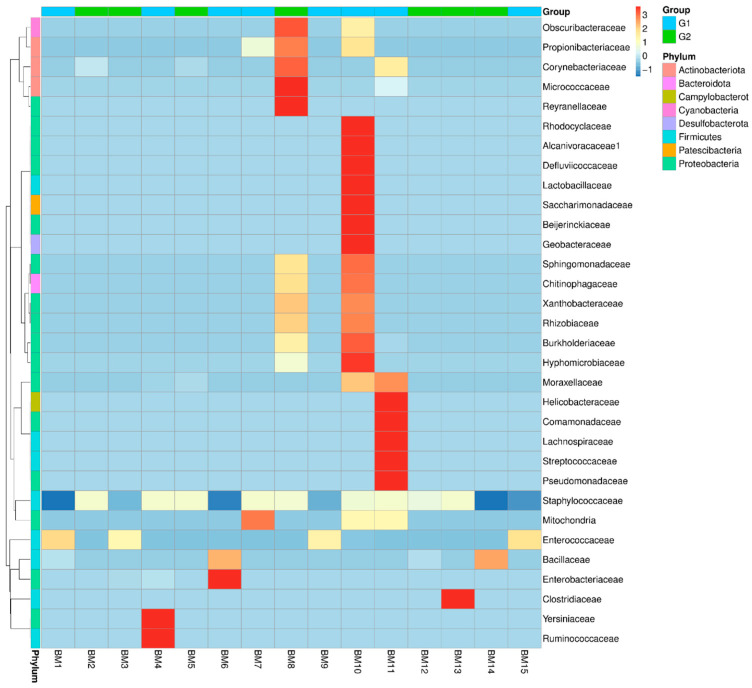
Taxonomic abundance cluster heatmap in different samples and groups at the family level. Values range from low (blue) to high (red).

Based on the abundance data for the top 35 genera across all samples, we generated a heatmap to examine whether samples with similar treatments cluster together, as well as to visualize their similarities and differences. The results are in Figure 2.

**Figure 3:**
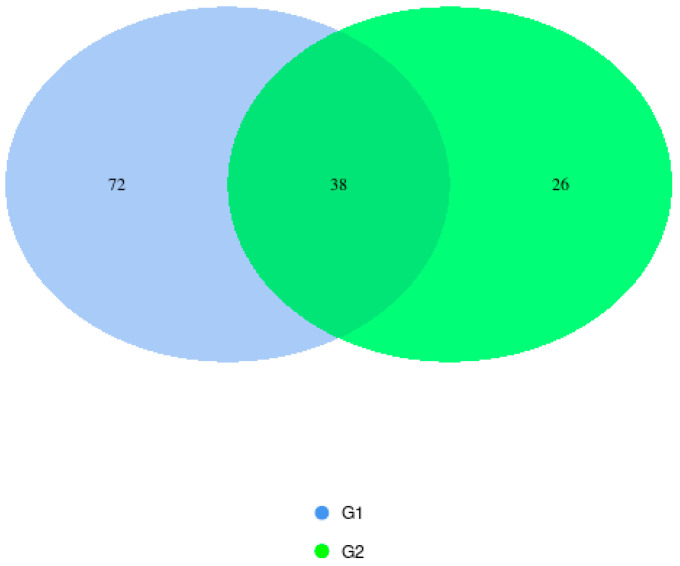
Venn diagram showing shared and unique OTUs between groups G1 and G2 after sequencing.

In the two birth-mode groups analyzed, sequencing revealed common phyla, including *Firmicutes*, *Cyanobacteria*, and *Proteobacteria*. Some phyla were found only in group G2, such as *Actinobacteria*. And some in group G1, such as *Bacteroidota*, *Desulfobacterota*, *Patescibacteria*, and *Campylobacterota*. In the group of vaginal births (G1), 72 OTUs were detected, and in the group of C-section births (G2), 26 units were detected. A much greater microbial diversity is observed in the colostrum of mothers who gave birth to their children vaginally. The results are presented in Figure 3.

The overlapping areas of the circles in the Venn diagram represent the core microbiome, which is generally defined as a shared group of microbiome members from similar habitats. Previous studies comparing the diversity and composition of the human colostrum microbiota in maternal cohorts of different ethnic backgrounds have also identified the most represented phyla as *Firmicutes* and *Proteobacteria* in samples from mothers who gave birth to their children vaginally [24]. Different studies have reported considerable variation in the bacterial diversity of colostrum, identifying as many as 465 genera, 645 species, and 7900 operational taxonomic units (OTUs) [25,26]. Culture-based assessments found bacterial loads ranging from 3.7 × 10^2^ to 1.1 × 10^9^ genome equivalents per milliliter [27,28], whereas qPCR analyses measured concentrations between 10^4^ and 10^6^ CFU/mL [29,30]. Khodayar-Pardo et al. found that colostrum contains the fewest bacteria overall, with total bacterial levels—and groups such as *Bifidobacterium* and *Enterococcus* species—increasing steadily throughout lactation, resulting in a richer microbial composition in mature milk [31]. Likewise, Boix-Amorós et al. reported that colostrum shows lower microbial diversity, which reaches its highest point in transitional milk. However, they also noted that *Acinetobacter* was more abundant in colostrum, whereas *Staphylococcus* remained the predominant genus at every stage of lactation [29]. Cabrera-Rubio et al. [10] found that colostrum contained higher levels of *Enterococcus*, while mature milk had greater amounts of *Staphylococcus aureus* and *Clostridium coccoides*, suggesting that the milk’s microbial profile changes as lactation progresses. The most dominant phyla found in colostrum were *Firmicutes* and *Proteobacteria*, with *Actinobacteria* and *Bacteroidetes* appearing in lower proportions [26,32,33]. Several additional phyla—such as *Acidobacteria*, *Armatimonadetes*, *Chlamydiae*, and *Verrucomicrobia*—were also detected. Across studies, commonly shared genera included *Bifidobacterium* spp., *Staphylococcus*, and *Streptococcus*, with *Bifidobacterium longum* and *Bifidobacterium breve* present in almost all samples [10,29]. Other frequently identified genera in colostrum were *Staphylococcus*, *Acinetobacter*, *Pseudomonas*, *Lactobacillus*, and members of *Clostridium*. Wyatt et al. noted a high abundance of *Streptococcus* and Micrococci, while Tang et al. and Obermajer et al. reported the presence of *Enterobacteriaceae*, including *Acinetobacter* and *Stenotrophomonas* [27,28,34].

**Figure 4:**
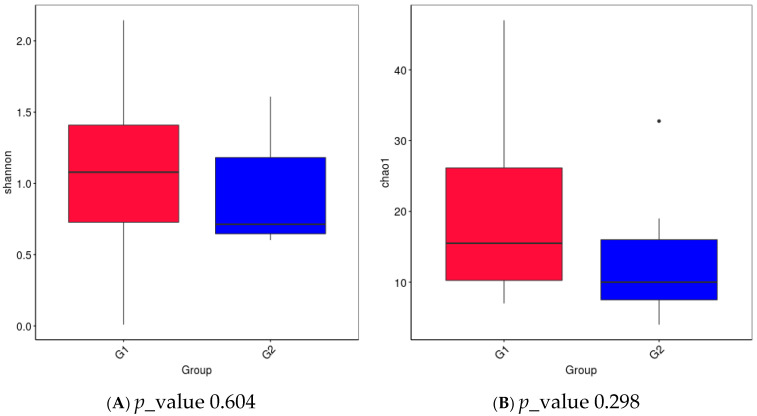
Alpha-diversity of the breast-milk microbiome in Group 1 (G1) and Group 2 (G2). Boxplots show (**A**) the Shannon diversity index and (**B**) the Chao1 richness estimator across samples from the two groups.

**Figure 5:**
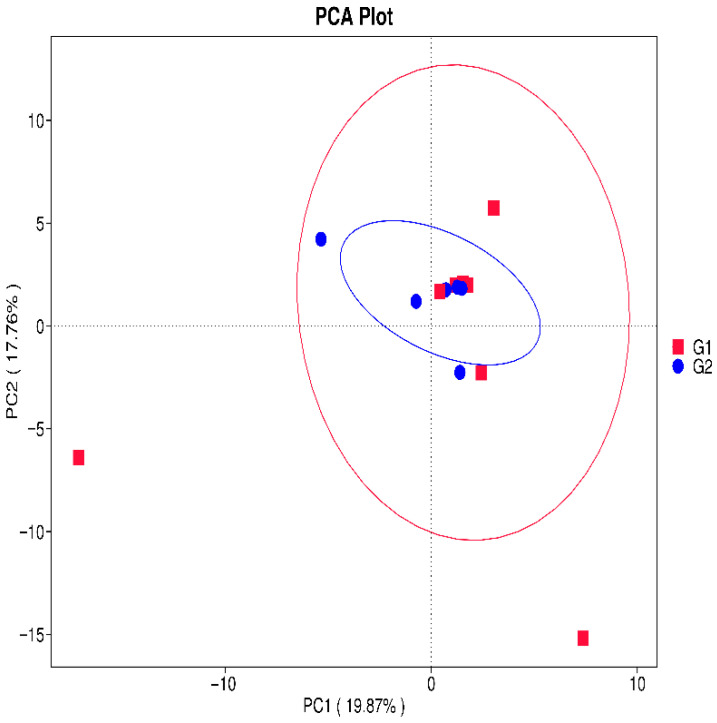
Principal Component Analysis (PCA) of Colostrum Microbiome Profiles by Delivery Mode.

In Figure 4, we presented the values of the Shannon index and Chao index for Alpha diversity. The Shannon diversity index was compared between Group 1 (G1) and Group 2 (G2). Overall, G1 exhibited a higher median alpha-diversity and a wider dispersion of values compared with G2, indicating greater microbial richness and/or evenness within this group. In contrast, G2 showed a lower central tendency and a narrower interquartile range, suggesting a more homogeneous and less diverse microbial community structure. This trend indicates that the breast-milk microbiome in G1 is compositionally more diverse than in G2. It is seen that both indices have a higher value in group G1, but there is no statistically significant difference between the two groups. In general, we can conclude that in group G1, the species diversity is greater and more evenly distributed, but without a statistical difference with group G2. It can be seen that the mode of birth does not lead to a statistical difference in the distribution of microbial species in colostrum. In some recent studies, other authors also found a lack of statistical difference between the two groups and a lack of influence of the birth method on both indices [35]. PCA was performed on genus-level relative abundance data to compare the microbial composition of colostrum from vaginal delivery mothers (G1, red squares) and C-section mothers (G2, blue circles). Each point represents an individual sample, and ellipses indicate the 95% confidence regions for each group. PC1 (19.87% of variance) and PC2 (17.76% of variance) together explain 37.63% of the total variability in the dataset. G1 samples show greater dispersion and several outliers, indicating higher intra-group microbial variability among vaginal deliveries. In contrast, G2 samples form a tighter cluster, reflecting a more homogeneous microbiome profile in C-section colostrum (Figure 5).

**Figure 6:**
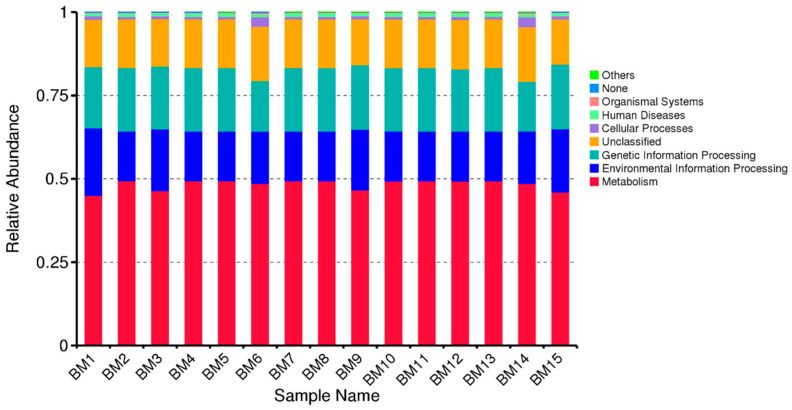
Predicted functional profile of the breast-milk microbiome across samples (BM1–BM15).

We used PICRUSt (Phylogenetic Investigation of Communities by Reconstruction of Unobserved States) to examine distinct microbiome groups and predict the functional potential of microbial communities based on gene sequencing data for 16S rRNA (Figure 6). Figure 6 suggests that the colostrum microbiome has a strong metabolic potential, which makes sense as microbes in colostrum can aid digestion and nutrient processing. Predictions are based on reference genomes, so unknown or unsequenced microbes may be misrepresented. The PICRUSt only predicts potential functions, not actual gene expression or activity. Bar charts show the relative abundance of predicted KEGG functional categories inferred from the microbial community composition. Across samples, functions associated with metabolism and environmental information processing represent the dominant functional groups, followed by genetic information processing and cellular processes. Minor contributions are observed for organismal systems and human disease–related pathways, while a small fraction of sequences remain unclassified. The functional distribution is broadly consistent across samples, indicating a relatively conserved metabolic and functional potential within the breast-milk microbiome.

**Figure 7:**
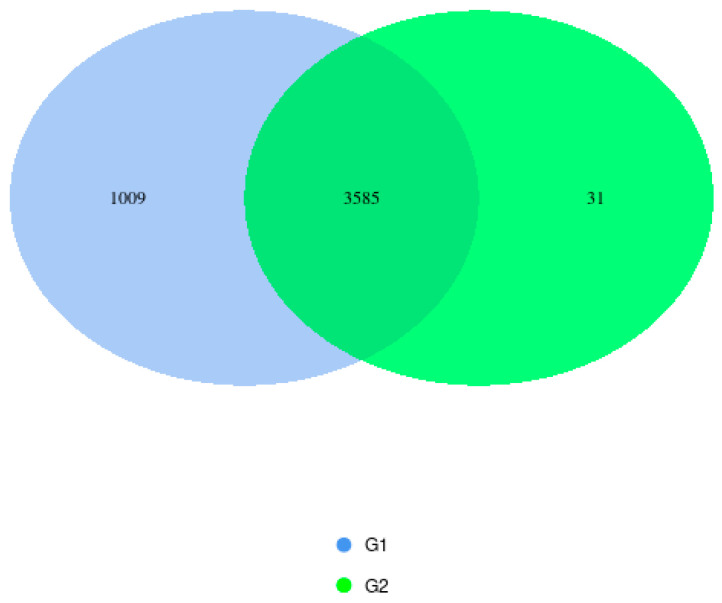
Shared and unique OTUs/ASVs between Group 1 (G1) and Group 2 (G2) of PICRUSt Results.

Figure 7 shows the distribution of predicted functional pathways between the two microbiome groups following PICRUSt analysis. A total of 3585 pathways were shared by both groups, representing the core predicted functional capacity. Group G1 contained 1009 unique pathways, indicating a higher functional diversity compared to G2, which exhibited only 31 unique pathways. These results suggest that while the two groups share a substantial functional core, G1 possesses additional specialized metabolic capabilities not found in G2. The predominance of shared taxa suggests a substantial common microbial community structure between groups, while the higher number of G1-specific taxa is consistent with the greater richness previously observed for this group.

**Figure 8:**
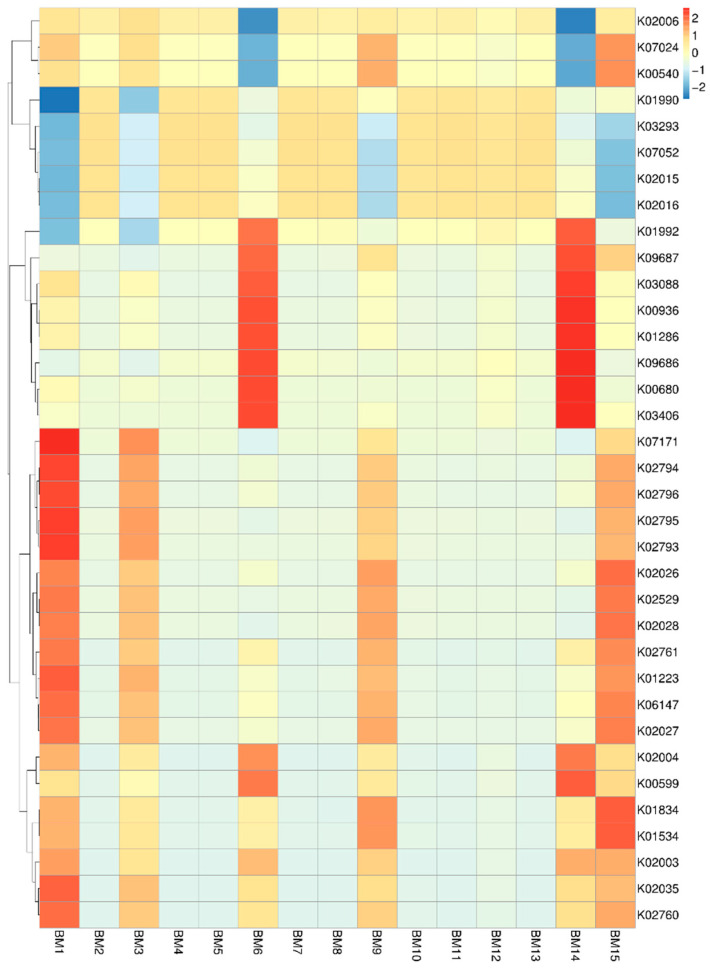
Heatmap showing the relative abundance of KEGG Orthology (KO) functional categories across samples.

The heatmap in Figure 8 shows substantial variation in functional gene abundance across breast milk samples collected from different mothers. Several KEGG Orthology (KO) groups display pronounced enrichment in specific samples—for example, BM1, BM6, and BM14—indicating that certain metabolic functions are more active in the microbiomes of some mothers than others. The heatmap shows the relative abundance of KEGG ortholog (KO) functions predicted from the breast-milk microbiome. Hierarchical clustering of samples and KOs reveals distinct functional patterns across individuals. Several clusters of enriched KOs correspond to pathways involved in carbohydrate metabolism, including enzymes associated with glycolysis and monosaccharide processing, which are more abundant in a subset of samples (e.g., BM3, BM6, BM10). Additional clusters include functions related to membrane transport and environmental information processing, particularly transporters and ABC-system components, suggesting variability in nutrient acquisition and microbial adaptation across samples. KOs linked to genetic information processing (e.g., replication and transcription machinery) and amino acid and energy metabolism were broadly detected at moderate levels across most samples, indicating a conserved functional core within the breast-milk microbiome. Despite inter-individual variation, these results suggest that functional redundancy is maintained, with variability driven primarily by metabolic and transport-associated pathways. This mother-to-mother variability suggests that the functional potential of the breast milk microbiome is not uniform but instead influenced by individual factors such as maternal physiology, lifestyle, diet, or environmental exposures. At the same time, many KOs remain consistently present at low or moderate levels across samples, reflecting a shared core set of microbial functions present in most mothers’ breast milk. Overall, the pattern highlights both a conserved functional baseline and notable inter-individual functional diversity among breast milk microbiomes.

## 4. Discussion

Across both birth-mode groups examined, sequencing revealed several shared phyla, including *Firmicutes*, *Cyanobacteria*, and *Proteobacteria.* However, certain phyla—such as *Chloroflexi* and *Actinobacteria*—were detected exclusively in group G2. In this study, we followed the composition of the microbiome in two separate groups of mothers and their colostrum. We also collected data on the mother’s age, gestational week, mode of delivery, and the newborn’s birth weight. As the most important factor for us that could determine the composition of the microbiome of the first milk, we considered that it could be the mode of delivery. Previous studies had found a statistically significant difference between the two groups of mothers and the breast milk microbiome [36,37]. For this reason, we focused on assessing the effect of this factor on the makeup of the colostrum microbiome. However, many of the studies had small sample sizes, and their findings differed across various populations and circumstances. As a result, it is essential to exercise caution when drawing firm conclusions about how these factors affect the composition of colostrum microbiota. Several explanations have been proposed for how microbes access and establish themselves in colostrum. One proposed route is retrograde flow, in which breast milk moves backward into the mammary ducts, carrying bacteria into the mammary gland [38]. Moreover, bacteria have been found in colostrum even before the infant begins nursing, suggesting that microbes are already present in the milk before the first feeding [39]. Maternal skin flora may also contribute to the microbial content of colostrum, as skin-related bacteria such as *Staphylococcus*, *Cutibacterium*, and *Corynebacterium* have been identified in breast milk Finally, the movement of bacteria from the gut to other parts of the body—a process that naturally increases during pregnancy and lactation in rodents—may also contribute to the presence of bacteria in colostrum. Together, these processes underscore the complex pathways through which microbes inhabit colostrum. Several studies have shown that colostrum contains a more diverse microbial community than mature breast milk. Khodayar-Pardo et al. reported that colostrum had higher microbial diversity, and Xie et al. observed greater microbial richness in colostrum as well, although they did not find significant differences in overall diversity indices. It would be interesting to track the microbiome of the first milk in mothers who gave birth to premature babies. Although some studies already have data on this influence. Asbury et al. observed that each mother’s milk contained distinct microbial communities, which underwent substantial changes over time. They also found that body mass index, mode of delivery, and, above all, the use of antibiotics during the prenatal period are associated with a significant influence on these microbial dynamics. Individual classes of antibiotics, as well as the duration of their use, showed unique relationships with the abundance and diversity of individual microbial taxa. These results highlight how the microbiota of mothers with premature babies evolves and stress the need for more in-depth studies on this topic.

## 5. Conclusions

In summary, colostrum harbors a highly diverse microbiota dominated by genera such as *Staphylococcus*, *Streptococcus*, *Lactobacillus*, *Bifidobacterium*, and *Enterococcus*. Across mothers, a shared core colostrum microbiome is present regardless of delivery mode, reflecting the contribution of maternal skin, mammary gland, and entero-mammary microbial reservoirs. However, our findings also indicate that the mode of delivery modulates specific features of this community. Vaginal delivery is generally associated with higher microbial richness and diversity, as well as a greater presence of taxa of vaginal and perineal origin, whereas cesarean delivery tends to result in a less diverse and more compositionally homogeneous microbiota, with a greater predominance of skin-derived bacteria. These patterns suggest that delivery mode does not replace the maternal core microbiome but rather shapes its structure and ecological variability.

Beyond delivery mode, the composition and diversity of colostrum microbiota are influenced by maternal characteristics (e.g., age, BMI, hormonal and secretory activity, stress, and gestational diabetes) and by external factors such as antibiotic exposure, diet, and geographic environment. The microbiota present in colostrum plays an essential role in the health of both infants and mothers: in infants, it contributes to early gut colonization, digestion, immune maturation, and protection against pathogens, partly through antimicrobial metabolites produced by lactic acid bacteria; in mothers, diverse milk microbiota may reduce the risk of mastitis and support mammary gland health.

Future research should include larger, methodologically robust studies that integrate delivery-mode stratification and longitudinal follow-up, and that aim to characterize the full microbial spectrum of colostrum—including fungi, archaea, and viruses—in order to better understand how these communities interact and how delivery mode influences their potential impact on infant development and maternal well-being.

## Figures and Tables

**Table 1:** Maternal and infant characteristics of the study participants.

Sample	Mother’s Age	Gestation Weeks	Birth Weight	Infant Sex	Delivery Mode
BM1	28	38	2920	Male	VD
BM2	41	37	3380	Male	CS
BM3	35	39	3740	Female	CS
BM4	36	37	2500	Female	VD
BM5	32	40	4020	Male	CS
BM6	36	37	2742	Male	VD
BM7	32	37	3080	Male	VD
BM8	21	38	3740	Female	CS
BM9	34	37	4100	Male	VD
BM10	29	40	3060	Female	VD
BM11	31	38	2900	Female	VD
BM12	21	38	3050	Male	CS
BM13	33	38	2840	Female	CS
BM14	26	39	2900	Male	CS
BM15	29	37	3230	Female	VD

## Data Availability

The original contributions presented in this study are included in the article/Supplementary Material. Further inquiries can be directed to the corresponding author.

## Supplementary Materials

The following supporting information can be downloaded at: https://www.mdpi.com/article/10.3390/microorganisms14010184/s1, Table S1: Functional Annotation of Transport Systems and Metabolic Enzymes (KEGG KO Entries).

## Abbreviations

The following abbreviations are used in this manuscript:

FAAs	Free amino acids
HM	Human milk
