# Supplementary material for: A Metagenomic Comparison of the Colostrum Microbiome in Bulgarian Mothers by Delivery Mode: A Pilot Study
Source: Microorganisms. 2026 Jan 14;14(1):184. doi: 10.3390/microorganisms14010184 (PMC12843876; doi:10.3390/microorganisms14010184)
Supplement: Supplementary file 1 [file microorganisms-14-00184-s001.zip › microorganisms-4041101-supplementary.pdf]

Table S1: Functional Annotation of Transport Systems and Metabolic Enzymes (KEGG KO Entries)

K02006	cobalt/nickel transport system ATP-binding protein
K07024	sucrose-6-phosphatase [EC:3.1.3.24]
K00540	F420H(2)-dependent quinone reductase [EC:1.1.98.-]
K01990	ABC-2 type transport system ATP-binding protein
K03293	amino acid transporter, AAT family
K07052	CAAX protease family protein
K02015	iron complex transport system permease protein
K02016	iron complex transport system substrate-binding protein
K01992	ABC-2 type transport system permease protein
K09687	sialidase-2/3/4 [EC:3.2.1.18]
K03088	RNA polymerase sigma-70 factor, ECF subfamily
K00936	sensor histidine kinase PdtaS [EC:2.7.13.3]
K01286	D-alanyl-D-alanine carboxypeptidase [EC:3.4.16.4]
K09686	small nucleolar RNA SNORD13
K00680	S-alkylcysteine N-acetyltransferase [EC:2.3.1.-]
K03406	methyl-accepting chemotaxis protein
K07171	mRNA interferase MazF [EC:3.1.-.-]
K02794	mannose PTS system EIIAB component [EC:2.7.1.191]
K02796	mannose PTS system EIID component
K02795	manY; mannose PTS system EIIC component
K02793	mannose PTS system EIIA component [EC:2.7.1.191]
K02026	Multiple sugar transport system permease protein
K02529	galR; LacI family transcriptional regulator, galactose operon repressor
K02028	polar amino acid transport system ATP-binding protein [EC:7.4.2.1]
K02761	cellobiose PTS system EIIC component
K01223	6-phospho-beta-glucosidase [EC:3.2.1.86]
K06147	ATP-binding cassette, subfamily B, bacterial
K02027	Multiple sugar transport system substrate-binding protein
K02004	putative ABC transport system permease protein
K00599	tRNA N(3)-methylcytidine methyltransferase METTL6 [EC:2.1.1.-]

K01834	2,3-bisphosphoglycerate-dependent phosphoglycerate mutase [EC:5.4.2.11]
K01534	Zn2+/Cd2+-exporting ATPase [EC:7.2.2.12 7.2.2.21]
K02003	putative ABC transport system ATP-binding protein
K02035	peptide/nickel transport system substrate-binding protein
K02760	cellobiose PTS system EIIB component [EC:2.7.1.196 2.7.1.205]
